# Serum IP-10 levels and increased DPPIV activity are linked to circulating CXCR3+ T cells in cholestatic HCV patients

**DOI:** 10.1371/journal.pone.0208225

**Published:** 2018-12-03

**Authors:** Monika Rau, Johannes Schmitt, Thomas Berg, Andreas E. Kremer, Bruno Stieger, Katharina Spanaus, Bertram Bengsch, Marta R. Romero, Jose J. Marin, Verena Keitel, Hartwig Klinker, Hans-Peter Tony, Beat Müllhaupt, Andreas Geier

**Affiliations:** 1 Division of Hepatology, Department of Medicine II, University Hospital Würzburg, Würzburg, Germany; 2 Division of Hepatology, Department of Gastroenterology and Rheumatology, University Hospital Leipzig, Leipzig, Germany; 3 Department of Medicine I, Friedrich Alexander University of Erlangen-Nuremberg, Erlangen, Germany; 4 Division of Clinical Pharmacology and Toxicology, University Hospital Zurich, Zurich, Switzerland; 5 Institute of Clinical Chemistry, University Hospital Zurich, Zurich, Switzerland; 6 Department of Medicine II, Gastroenterology, Hepatology, Endocrinology, and Infectious Diseases, University Medical Center Freiburg, Germany; 7 BIOSS Centre for Biological Signaling Studies, Freiburg, Germany; 8 Laboratory of Experimental Hepatology and Drug Targeting, CIBERehd, IBSAL, University of Salamanca, Salamanca, Spain; 9 Clinic for Gastroenterology, Hepatology and Infectious Diseases, University Hospital Düsseldorf, Medical Faculty at Heinrich-Heine-University, Düsseldorf, Germany; 10 Division of Infectious Disease, Department of Medicine II, University Hospital Würzburg, Würzburg, Germany; 11 Division of Rheumatology, Department of Medicine II, University Hospital Würzburg, Würzburg, Germany; 12 Department of Gastroenterology and Hepatology, University Hospital Zurich, Zurich, Switzerland; Texas A&M University, UNITED STATES

## Abstract

**Background & aims:**

Serum interferon-gamma-inducible protein-10 (IP-10) is elevated in cholestatic liver diseases and predicts response to antiviral therapy in patients with chronic hepatitis C virus (HCV) infection. Dipeptidylpeptidase 4 (DPPIV) cleaves active IP-10 into an inactive form, which inhibits recruitment of CXCR3+ T cells to the liver. In this study the link between IP-10 levels, DPPIV activity in serum and CXCR3+ T cells is analysed in cholestatic and non-cholestatic liver patients.

**Methods:**

In serum DPPIV activity (by enzymatic assay), IP-10 (by ELISA) and bile acids (BA) (by enzymatic assay) were analysed in 229 naive HCV genotype (GT) 1 patients and in 16 patients with cholestatic liver disease. In a prospective follow-up (FU) cohort of 27 HCV GT 1 patients peripheral CD3+CXCR3+, CD4+CXCR3+ and CD8+CXCR3+ cells were measured by FACS.

**Results:**

In 229 HCV patients serum IP-10 levels correlated positively to DPPIV serum activity. Higher IP-10 levels and DPPIV activity were detected in cholestatic and in cirrhotic HCV patients. Increased IP-10 serum levels were associated with therapeutic non-response to antiviral treatment with pegylated-interferon and ribavirin. In the HCV FU cohort elevated IP-10 serum levels and increased BA were associated with higher frequencies of peripheral CD3+CXCR3+, CD4+CXCR3+ and CD8+CXCR3+ T cells. Positive correlation between serum IP-10 levels and DPPIV activity was likewise validated in patients with cholestatic liver diseases.

**Conclusions:**

A strong correlation between elevated serum levels of IP-10 and DPPIV activity was seen in different cholestatic patient groups. Furthermore, in cholestatic HCV patients a functional link to increased numbers of peripheral CXCR3+ immune cells could be observed. The source of DPPIV release in cholestatic patients remains open.

## Introduction

Cholestasis is characterized by elevated hepatic and serum bile acids (BA) and may lead to changes in liver tissue such as acute hepatocellular toxicity, bile duct proliferation and fibrosis progression up to biliary cirrhosis [1]. High serum BA levels are a hallmark in cholestatic liver diseases with obstructive jaundice and likewise in primarily noncholestatic liver diseases such as chronic hepatitis C virus (HCV) infection [2, 3]. In HCV patients, increased BA serum levels have been previously associated with a non-response to former antiviral treatment with pegylated interferon (Peg-IFN) and ribavirin (RBV) and were proposed as predictors for the severity of liver fibrosis [3–5]. We recently described an association between genetic predisposition for cholestasis with a polymorphism in the gene coding for the bile salt export pump (BSEP; ABCB 11 1331C allele) and non-sustained virological response (SVR) in a HCV patient cohort [6]. In the same work patients with non-achieving SVR had significantly higher serum BA levels than those with SVR. Furthermore, increased BA levels are associated to direct acting antiviral treatment with ombitasvir/paritaprevir/ritonavir ± dasabuvir in a recent preliminary study of 20 HCV patients probably due to alterations of bile acid transport [7].

Higher expression of the chemokine interferon γ inducible protein 10 (IP-10/CXCL10) in liver and peripheral blood is described in patients with chronic HCV infection and are associated with liver fibrosis [8, 9]. In HCV patients pretreatment levels of IP-10 are a good predictive marker for therapy outcome and correlate to non-response to antiviral treatment [10–13]. Additionally, changes of serum IP-10 in HCV patients treated with sofosbuvir and ribavirin mirrored HCV RNA and seemed to be an indicator of innate immune viral recognition [14].

T cell migration into the liver is conducted by chemokine production in liver tissue, e.g. IP-10 and monokine induced by interferon-gamma (MIG/ CXCL9). Both chemokines are preferentially expressed by sinusoidal epithelium and recruit T cells into the liver by binding to CXCR3, a receptor that is upregulated on activated lymphocytes [12]. Thus, IP-10 is an important chemokine for selective immune cell trafficking to the liver by binding to CXCR3 [8]. The NH_2_-terminal region of chemokines is crucial for receptor binding and signaling activities and the activity of chemokines can be regulated by post-translational modification [15]. N-terminal removal of two amino acids of active IP-10 (aIP-10) by dipeptidyl peptidase 4 (DPPIV/ CD26) leads to an antagonist form (cIP-10) [15, 16]. In the study of *Casrouge et al*. the antagonist form of IP-10 (cleaved IP-10 (cIP-10)) was elevated in HCV patients especially in patients with non-response to antiviral treatment. [16]. The antagonist form of IP-10 (c-IP10) still binds to CXCR3 receptor but without induction of signaling and therefore without recruitment of immune cells into the liver. The authors concluded that the dominant form of IP-10 in HCV patients is the antagonist form and this chemokine antagonism may contribute to treatment failure due to an impaired immune cell recruitment into the liver [16]. In consequence, a higher abundance of these immune cells remains detectable in the peripheral blood. Furthermore, a recent study demonstrated that the truncation of IP-10 alters lymphocyte migration and limits infiltration of tumor parenchyma in mice and can be reversed through the DPPIV inhibitor sitagliptin [17]. It is challenging to study post-transcriptional modifications of chemokines in biological samples, as it is difficult to monitor the different protein forms.

The serine protease DPPIV is present on different cell types, e.g. the canalicular membrane of hepatocytes, the apical membrane of cholangiocytes, and lymphocytes. Elevated serum activity is described in patients with cholestatic liver disease such as biliary atresia, primary biliary cirrhosis, in HCV patients as well as in animal models with bile duct ligation [18–20].

From the above described data, we hypothesized that high serum BA favors release of DPPIV and subsequently an increase in serum levels of IP-10 (cIP-10), which leads to a reduced infiltration of CXCR3 positive cells into the liver. Finally, these events could account for the unfavorable effect of reduced viral clearance in HCV patients, but also have clinical impact on infectious complications in other cholestatic liver diseases.

The aim of this study was to analyse IP-10 serum levels as well as DPPIV activity in cholestatic HCV patients. The influence of IP-10 levels and DPPIV activity on circulating immune cells was investigated in a smaller follow-up cohort of HCV infected patients. Findings were furthermore validated in 16 cholestatic patients with other underlying cholestatic liver diseases.

## Methods

### Patient cohorts

#### Hepatitis C patients

229 patients with HCV GT 1 infection were included in this study after written informed consent. 48 patients from the Swiss Hepatitis C Cohort Study (SCCS) and 181 patients from the Berlin cohort were recruited between 2000 and 2008. The study was approved by the local ethics committees (University Hospital Zürich EK-695; Berlin cohort AZ205/2002). Response to antiviral treatment with Peg-IFN and RBV was documented by SVR and Non-SVR including relapse, partial response and non-response to antiviral treatment. Serum samples were collected before antiviral treatment and stored immediately at -80°C.

#### Hepatitis C follow-up (FU) cohort and healthy controls

Serum samples and peripheral blood mononuclear cells (PBMC) were collected in 27 HCV GT 1 patients and 17 healthy controls (HC) after approval by the local ethics committees (University Hospital Würzburg AZ53/12) and given written informed consent. HCV patients in this cohort were either treatment-naive or showed relapse to a previous antiviral treatment. HC had no evidence of any liver disease by medical history and questionnaire.

#### Cholestatic non-HCV validation cohort

Six patients with primary biliary cirrhosis (PBC), six patients with primary sclerosing cholangitis (PSC) and 4 patients with bile duct drainage were included in this study after written informed consent and approval by local ethics committees (University Hospital Erlangen AZ238_13B) Serum BA were measured before (mean = 114 ± 104 μM) and after treatment with cholestyramine in PBC/PSC patients for 2 to 3 weeks (mean = 90 ± 140 μM). Serum samples of patients with biliary drainage were available at day 0 and at day 3 after bile duct drainage (Table 1). 3 out of 4 patients had decrease in serum BA, whereas 1 patient had increase in serum BA on day 3.

**Table 1:** Patient characteristics.

Patient characteristics % (n)	
***Hepatitis C patients***	n = 229
**sex m/f**	62.4 (143) / 37.6 (86)
**cirrhosis/non-cirrhosis/missing**	16.6 (38)/ 74.7 (171)/ 8.7 (20)
**SVR/no SVR**	34.5 (79)/ 65.5(150)
**BA elevated/non-elevated**	40.6 (93)/ 59.4 (136)
***Hepatitis C follow-up cohort***	n = 27
**sex m/f**	59.3 (16) / 40.7 (11)
**cirrhosis/non-cirrhosis**	22.2 (6)/ 77.8 (21)
**treatment-naiv/no SVR**	63.0 (17)/ 37.0 (10)
**BA elevated/non-elevated**	11.1(3)/ 88.9 (24)
***Cholestatic non-HCV patients***	n = 16
**sex m/f**	31.3 (5)/68.9 (11)
**PSC/PBC/biliary drainage**	37.5 (6)/ 37.5 (6)/ 25 (4)

### IP-10 measurement

Quantitative determination of human IP-10 (overall IP-10, no differentiation of aIP-10 or cIP-10) in serum was carried out using the HK311 human IP-10 ELISA KIT (Hycult biotech, PA). Measurable concentration range is from 20 to 5,000 pg/mL. Serum samples were diluted (1:4) as described in the protocol and the absorbance was measured at 450 nm.

IP-10 levels in serum samples of the FU cohort (27 patients with chronic HCV infection and 17 HC) were measured as overall IP-10, aIP-10 and c-IP10 by diagnostic lab, Myriad-Rules Based Medicine (Austin, USA). In 18 patients overall IP-10 was under the lower limit of quantification of this assay. These patients were included in the analysis with the value of the lower limit of quantification (212 pg/mL). In the cholestatic non-HCV validation cohort (n = 16) IP-10 serum concentration was measured by using the Human CXCL10/IP-10 Quantikine ELISA kit (R&D Systems, Minneapolis, USA) measuring overall IP-10 (no differentiation of aIP-10 or cIP-10). The ELISA was performed according to the manufacturer’s instructions.

### Serum DPPIV activity

Enzymatic activity of DPPIV was measured in serum by a chromogenic substrate (H-Gly-Pro-pNA) (Enzo Life science, Farmingdale, NY). Cleavage of the p-nitroaniline (pNA) from the chromogenic substrate increases absorbance at 405 nm.

### Flow cytometry of PBMC

100 μl of peripheral patient blood were incubated with 10 μl of antibody (CD3 PC5 Beckman Coulter (Beckman Coulter, Brea, CA), CD4 ECD Beckman Coulter and CXCR3 Alexa Fluor 488 Biolegend (Biolegend, San Diego, CA) and thereafter VersaLyse (Beckman Coulter, Brea, CA) was added. After standard washing procedure stained cells were analysed and measured by flow cytometry.

### Bile acid measurement

Serum BA levels in 256 patients were analysed as described previously [21]. 100 μl of serum were diluted by 100 μl isotonice saline, BA measurement was performed enzymatically with spectrophotometric detection (Trinity Biotech, Wicklow, Ireland). The upper reference limit was 8 μM. Serum BA levels were considered elevated above 10μM, as this this cut-off is considered clinically relevant for the risk assessment to predict complications in intrahepatic cholestasis of pregnancy. Total serum BA levels in the cohort of 16 cholestatic patients were quantified using Diazyme total bile salts kit (Diazyme Laboratories, Poway, CA) according to the manufacturer’s instructions.

### Statistical analysis

Statistical analyses were performed using SPSS (19.0, SPSS Inc., Chicago) and graphs were created with Prism5 (GraphPad Software, La Jolla, CA).

The independent, unpaired t-test or the non-parametric Wilcoxon-Mann-Whitney-test (group with n ≤ 10 or missing normal distribution) was used as appropriate to analyse differences between groups. Correlation was calculated by using Spearman-Rho or Pearson-Rho in non-parametric or normal distributed variables as appropriated. A P-value less than 0.05 were considered statistically significant (*), p < 0.01 = (**), p < 0.001 = (**).

## Results

### Serum IP-10 and DPPIV activity are correlated to cholestasis and cirrhosis in HCV patients

IP-10, BA levels and DPPIV activity in serum were analysed in a cohort of 229 naive HCV-patients. Detailed patient characteristics are depicted in Table 1.

**Fig 1:**
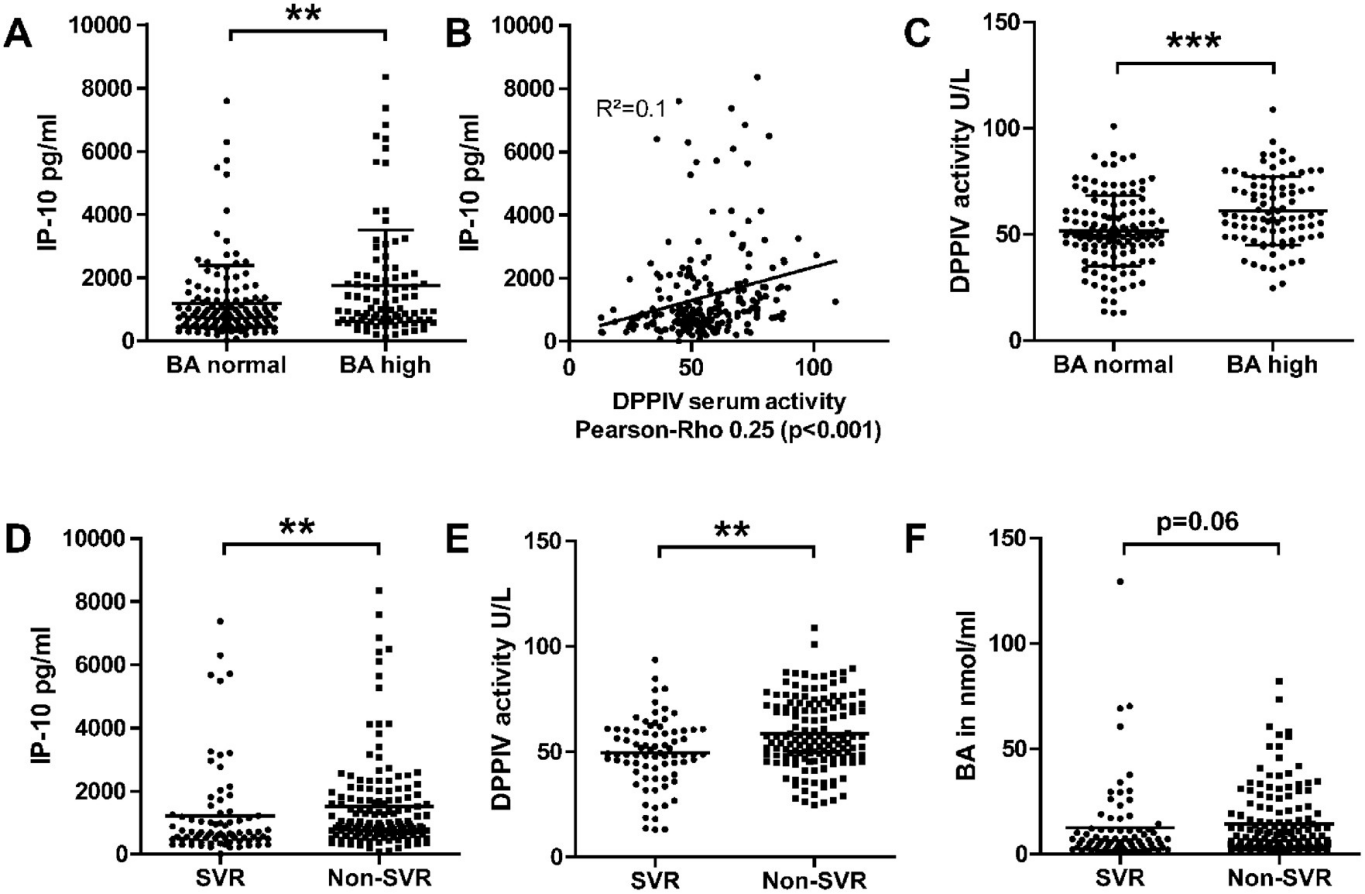
Serum IP-10 levels, DPPIV activity and BA levels in HCV patients (n = 229). A) Cholestatic HCV patients with serum BA > 10μM had higher IP-10 serum levels. Mean ± SD (Wilcoxon Mann Whitney test; ** = p<0.01). B) Positive correlation between IP-10 serum levels and DPPIV serum activity (Pearson-Rho 0.25; p<0.01). C) Cholestatic HCV patients had higher DPPIV serum activity. Mean ± SD (Wilcoxon Mann Whitney test; *** = p<0.001). D) Non-SVR patients had higher IP-10 levels (E), higher DPPIV serum activity (F) and a trend towards higher serum BA compared to HC. Mean ± SD (Wilcoxon Mann Whitney test; ** = p < 0.01).

Cholestasis was defined as elevated serum BA concentration > 10μM. The cut-off level of 10μM was chosen as this cut- off is considered clinically relevant for the risk assessment to predict complications in intrahepatic cholestasis of pregnancy [22]. 93 of 229 patients (40.6%) had elevated serum BA before antiviral treatment (Table 1) and these patients showed also significantly higher IP-10 serum levels compared to patients without cholestasis (Fig 1A). IP-10 is undergoing a post-translational modification with a N-terminal cleavage by DPPIV leading to a truncated form (antagonist form of IP-10 (cIP-10)) that binds to CXCR3 receptor, but does not induce cellular signalling and thereby leads to a 10-fold reduced chemotactic potency [15]. In our large cohort of HCV patients, DPPIV serum activity and serum IP-10 levels (no differentiation between aIP-10 and cIP-10) showed a positive correlation (Pearson-rho 0.25 (p<0.001) (Fig 1B). Interestingly, cholestatic HCV patients (serum BA concentration > 10 μM) had a significantly higher enzymatic activity of DPPIV in serum compared to non-cholestatic HCV patients (Fig 1C).

Furthermore, higher IP-10 serum levels were associated with impaired therapy response in this cohort of HCV patients. Patients with non-SVR had significantly higher serum IP-10 levels before treatment as compared to patients with SVR (Fig 1D). In line with this finding non-SVR patients had also significantly higher DPPIV serum activity as well as a trend towards elevated serum BA in comparison to HC (Fig 1E and 1F).

**Fig 2:**
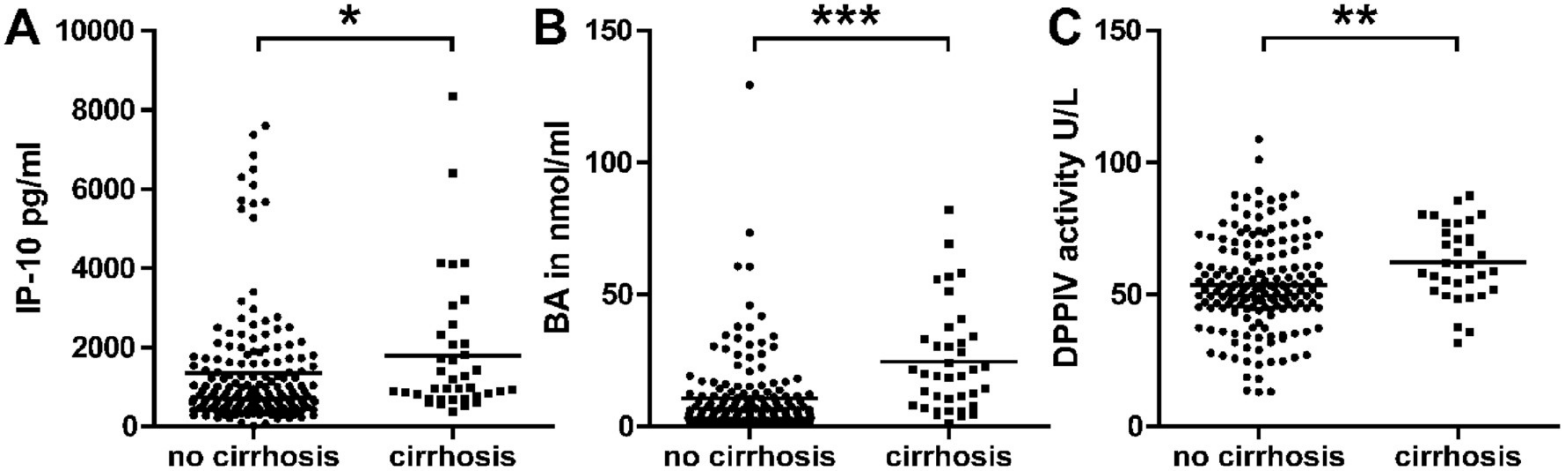
HCV patients with cirrhosis. HCV patients with cirrhosis (n = 38) had significantly higher IP-10 and BA serum levels as well as DPPIV serum activity compared to patients without cirrhosis. Total HCV cohort n = 229. Mean ± SD. * = p < 0.05; ** = p < 0.01; *** = p < 0.001 (Wilcoxon Mann Whitney test).

In cirrhotic patients (n = 38) significant higher serum BA (mean ± SEM: 25.2 ± 3.4 μM) were observed compared to non-cirrhotic patients (n = 171) (10.8 ± 1.1 μM, p < 0.001). These patients with advanced disease showed also significantly higher IP-10 serum levels as well as DPPIV serum activity as depicted in Fig 2A–2C.

### Peripheral CXCR3+ T cells are linked to higher serum IP-10 in cholestatic HCV patients

**Fig 3:**
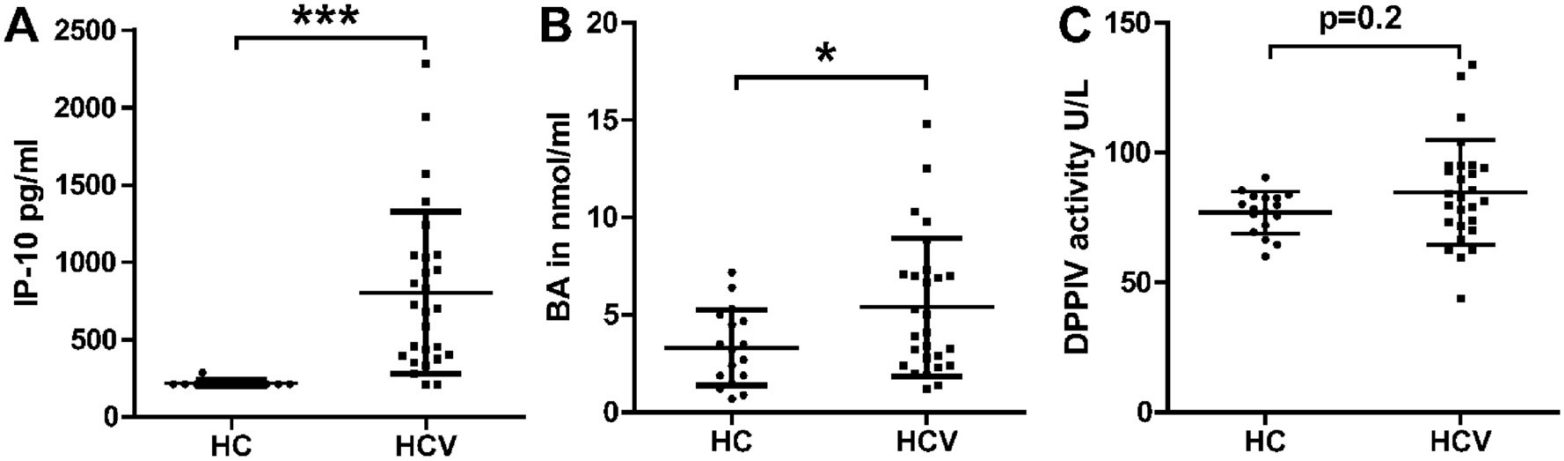
Serum IP-10, BA and DPPIV activity in a prospective FU cohort. 27 HCV patients IP-10 levels as well as BA levels were significantly higher compared to HC. DPPIV serum activity was higher in HCV patients, but without reaching statistically significance. Mean ± SD. (Wilcoxon Mann Whitney test) *** = p < 0.001; * = p < 0.05.

In a prospective follow-up (FU) cohort of 27 HCV GT 1 patients and 17 HC PBMC were analysed together with above described serum markers to investigate the mechanistic relationship between serum IP-10 levels and CXCR3+ T cells. Clinical characteristics are depicted in Table 1. Serum IP-10 and BA concentrations were significantly higher in HCV patients compared to HC. DPPIV serum activity showed no significant difference between HCV patients and HC (Fig 3A–3C).

**Fig 4:**
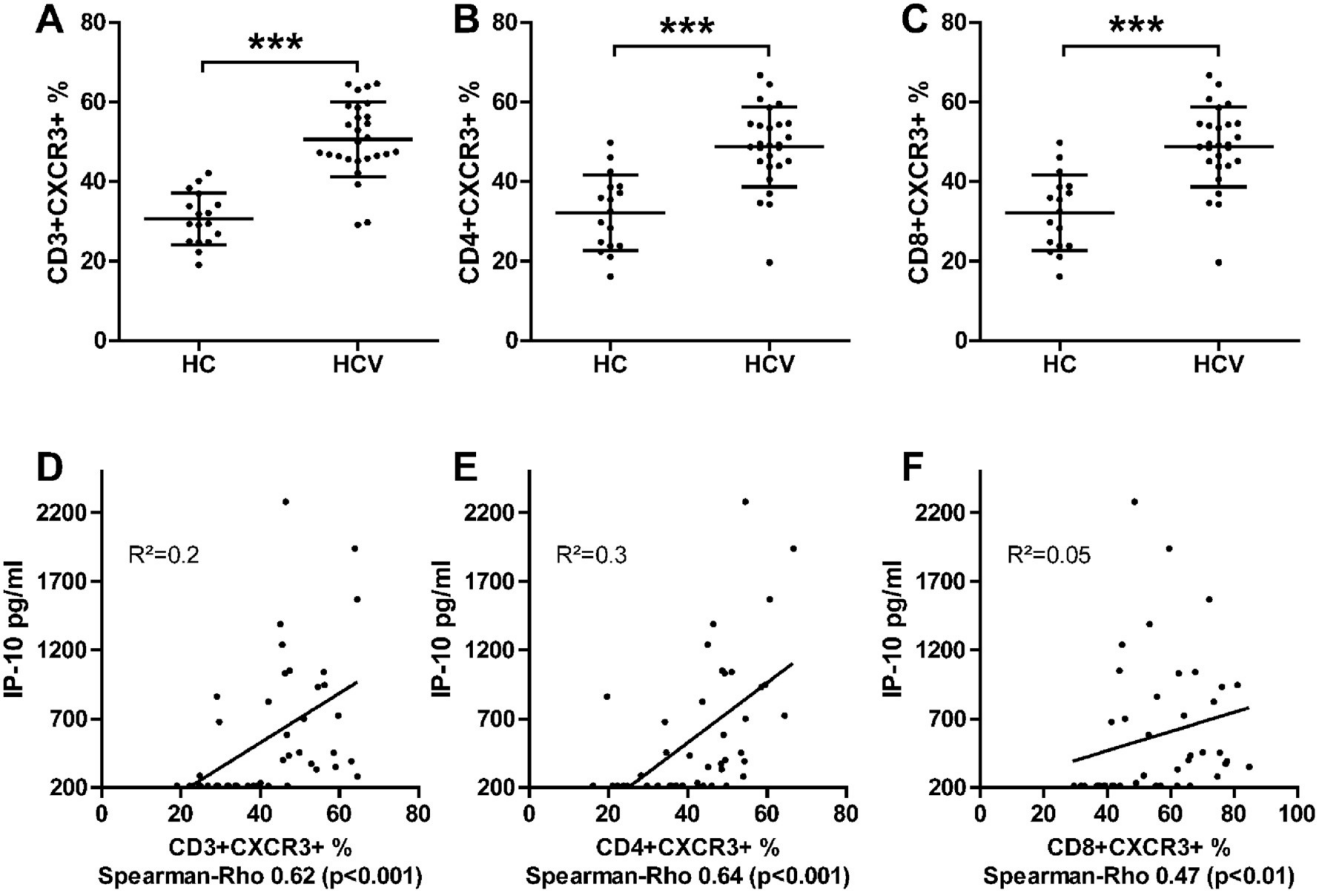
Immmunological data of FU cohort with 27 HCV patients and 17 HC. HCV patients had higher frequency of (A) CD3+CXCR3+, (B) CD4+CXCR3+ and (C) CD8+CXCR3+ T cells in peripheral blood. Mean ± SD. (Wilcoxon Mann Whitney test) *** = p < 0.001; ** = p < 0.01; * = p < 0.05. D-F) Correlation between serum IP-10 levels and peripheral (D) CD3+CXCR3+, (E) CD4+CXCR3+ and (F) CD8+CXCR3+ T cells. Spearman Rho as indicated.

The chemokine IP-10 binds to CXCR3, a receptor that is expressed on activated lymphocytes. In this FU cohort peripheral CD3+CXCR3+, CD4+CXCR3+ and CD8+CXCR3+ cells were analysed. HCV patients had significantly more CD3+CXCR3+, CD4+CXCR3+ and CD8+CXCR3+ cells compared to HC (HCV vs. healthy controls; mean ± SEM CD3+CXCR3+: 50.6 ± 1.8 vs. 30.5 ± 1.6%, CD4+CXCR3+: 48.7 ± 1.9 vs. 32.1 ± 2.3% and CD8+CXCR3+: 63.7 ± 2.4 vs. 42.6 ± 2.3%) (Fig 4A–4C). Furthermore, a positive correlation was observed for IP-10 serum levels and CD3+CXCR3+ (Spearman-Rho 0.62; p<0.001), CD4+CXCR3+ (Spearman-Rho 0.64; p<0.001) and CD8+CXCR3+ T cells (Spearman-Rho 0.47; p<0.01) (Fig 4D–4F).

In this small FU cohort, the above described analyses in the large cohort were repeated and are depicted in S1 Fig. Only three patients were cholestatic in the FU cohort. These patients had higher IP-10 serum levels, but no significant difference was observed in DPPIV serum activity compared to individuals without cholestasis. Cirrhotic patients (n = 6) had significantly higher IP-10 serum levels as well as BA levels compared to non-cirrhotic patients. For DPPIV serum activity no significant difference between cirrhotic and non-cirrhotic patients was observed in this small cohort. Further BA differentiation has been performed by gas chromatography-mass spectrometry HPLC-MS/MS. Taurine-conjugated BA were significantly elevated an associated to higher IP-10 serum levels in HCV patients (S2 Fig).

In this FU cohort serum IP-10 levels were analysed by diagnostic lab (Myriad RBM) to differentiate cIP-10 from agonist IP-10 (aIP-10). But aIP-10 was only detected in two HCV patients and cIP-10 only in three patients and four HC. Therefore, no in-depth analysis could be performed with these data (**data not shown**).

Immune cell changes were also analysed in cholestatic as well as in cirrhotic patients in the FU cohort. Three cholestatic patients showed a trend towards higher CD3+CXCR3+ T cells in peripheral blood and six cirrhotic patients had a trend towards higher frequency of CD4+CXCR3+ T cells (S3 Fig). One could speculate that due to the low number of cholestatic and cirrhotic patients these results did not reach significance.

### Cholestatic non-HCV patients showed as validation cohort a strong interplay between serum IP-10 levels and DPPIV serum activity

In this patient group PBC, PSC as well as patients with biliary drainage were included. Six patients with PBC and six patients with PSC received treatment with cholestyramine against pruritus for 2–3 weeks. Eight patients had a decrease in serum BA during cholestyramine treatment (relative change mean ± SD: -51.5 ± 22.7%) and four patients had an increase in serum BA (relative change: +86.1 ± 47.0%). Three of four patients with biliary drainage had a decrease in serum BA and one patient showed increased serum BA on day three after drainage.

**Fig 5:**
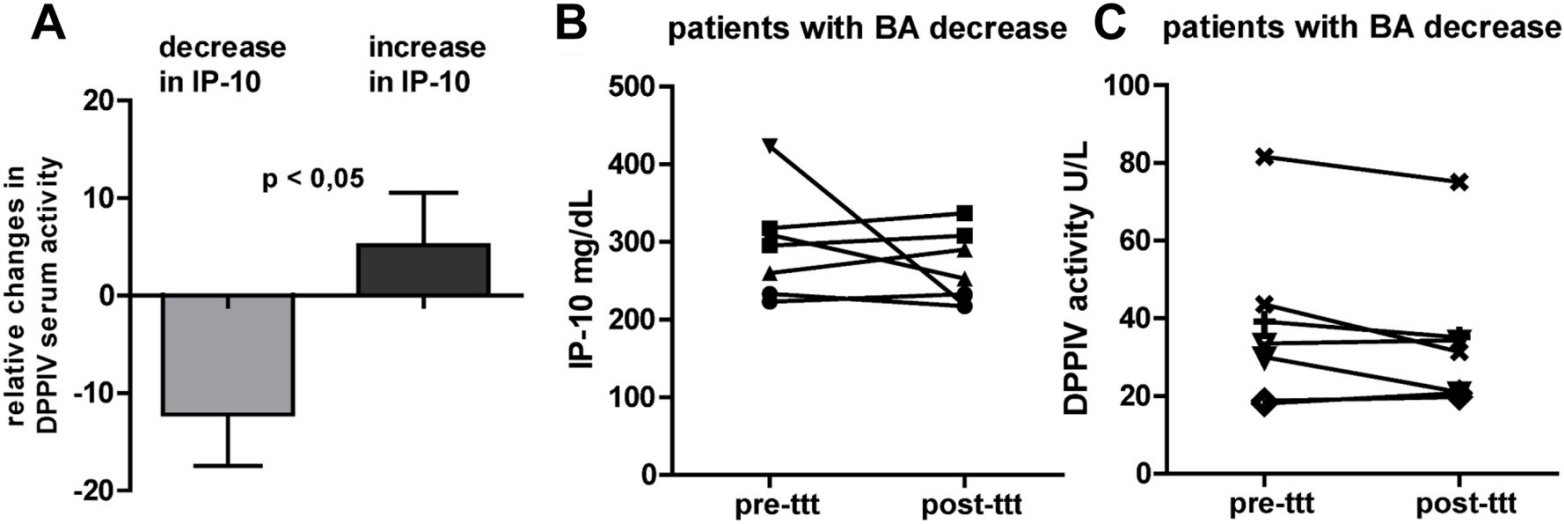
Validation cohort with cholestatic liver disease (PBC/PSC) and biliary drainage (n = 16). A) Patients with relative decrease/increase in IP-10 had significantly relative decrease/increase in DPPIV serum activity. B-C) Seven representative patients with highest decrease in BA and the corresponding (B) serum IP-10 and (C) DPPIV activity over time.

In this validation cohort a positive correlation between relative changes in DPPIV serum activity and IP-10 levels over time was observed (Rho pearson 0.468, p < 0.05). Cholestatic patients with relative increase in IP-10 serum levels over time had significantly more often an increase in relative changes of DPPIV serum activity (Fig 5A). Seven representative patients with the highest decrease of serum BA over time are depicted in Fig 5B. No homogenous trend towards decrease in IP-10 serum levels and DPPIV activity before and after treatment with cholestyramine or biliary drainage was observed.

## Discussion

Cholestasis with elevated serum BA is a hallmark in chronic liver disease. In the present study serum markers such as IP-10 and DPPIV activity were analysed in cholestatic HCV patients to investigate a possible linkage between IP-10, DPPIV and cholestasis. Higher IP-10 serum levels can be observed in different liver pathologies, e.g. viral hepatitis B/C, PBC, biliary atresia and autoimmune liver disease with normalisation after treatment response [10, 11, 13, 23–25]. In our large cohort of 229 HCV patients higher IP-10 serum levels were observed in cholestatic HCV patients as well as in patients with therapeutic non-response to antiviral treatment and IP-10 serum levels were correlated to higher serum DPPIV activity (Fig 1). But in the above-mentioned studies no information about serum BA as hallmark of progressive liver disease has been reported. In our study analysis of serum BA was performed and a significant association between higher IP-10 serum levels and higher serum BA was described for the first time in cholestatic HCV patients (Fig 1).

*Casrouge et al*. first described higher levels of IP-10 antagonist form (cIP-10) after cleavage by DPPIV in 34 patients with chronic HCV infection. Higher levels were measured in treatment non-responders as compared with responders. Furthermore in 5 patients initiating antiviral treatment with PEG-IFN and RBV, decreased number of CXCR3 expressing cells were detected in the circulation 6 hours after first dosage together with higher IP-10 serum levels [16]. In our FU study analyses of CD3+CXCR3+, CD4+CXCR3+ and CD8+CXCR3+ cells were performed and a significant higher frequency of CXCR3 expressing T cells was detected in patients with chronic HCV infection (n = 27) (Fig 4A–4C). Furthermore, higher IP-10 serum levels were correlated to higher frequency of CD3+CXCR3+, CD4+CXCR3+ and CD8+CXCR3+ cells (Fig 4D and 4E). These correlations indicate the disturbed homing of CXCR3+ T cells in HCV patients by post-transitional modified IP-10. In line with our hypothesis, that cholestasis could lead to a higher DPPIV serum activity and therefore higher antagonist form of IP-10 (cIP-10), three HCV patients with increased serum BA showed a trend toward higher frequency of CD3+CXCR3+ T cells in peripheral blood. Due to the low patient number no statistical significance was reached (S3 Fig).

It is challenging to study post-transcriptional modifications of chemokines (such as aIP-10 and cIP-10) in biological samples, as it is difficult to monitor the different protein forms [26]. In a cohort of 69 Egyptian patients with chronic HCV GT 4 infection IP-10 agonist (aIP-10) and antagonist (cIP-10) forms were analysed, but the antagonist form (cIP-10) was not detected with the exception of three patients [27]. In our prospective FU cohort, the same assay was performed but the antagonist (cIP-10) form was not detected with the exception of three patients and four HC (data not shown) In recent studies a novel ultrasensitive single-molecule assay (Simoa) was used to distinguish aIP-10 and cIP-10 [26, 28]. One of these studies analysed the effects of DPPIV inhibition in healthy controls and HCV patients. The treatment with 100mg sitagliptin preserved the bioactive form of IP-10 (aIP-10) [26]. In a recent study *Blauenfeldt* et al. analysed the homing effect of CXCR3+ T cells by IP-10 in patients with tuberculosis. In this study, patients with active tuberculosis had high levels of cIP-10 and a reduced frequency of CXCR3+ T cells was observed at the pulmonal site of infection compared to peripheral blood. This finding reflects a possible regulatory role of DPPIV in homing of CXCR3+ T cells to the site of infection [29]. This finding is in line with our hypothesis, that cIP-10 (antagonist form) leads to an inefficient homing of CXCR3+ T cells at the site of infection (e.g. lung for tuberculosis or in the present study liver for HCV infection).

The origin of DPPIV is still not properly understood. However, there are different studies suggesting the hepatobiliary system as primary source because of a strong correlation between liver enzymes and DPPIV serum levels in patients with different liver pathologies (e.g. viral hepatitis, non-alcoholic fatty liver disease, hepatocellular carcinoma and cirrhosis) [30, 31]. DPPIV was also proposed as a serum marker for cholestasis since good correlation to serum bilirubin was described [20]. But in these studies, no measurement of serum BA as hallmark of chronic liver disease was performed. Activated T cells and especially Th17 cells show high expression of CD26/DPPIV and could likewise be source of higher DPPIV activity [32].

To underline our findings independently of HCV infection DPPIV serum activity, IP-10 serum levels and serum BA were quantified in a cohort of cholestatic patients with PSC, PBC or biliary drainage. Similar, in this non-HCV cohort DPPIV serum activity correlated with IP-10 serum levels (Fig 5A). However, no strong correlation between serum IP-10/DDPIV and BA was observed in this non-viral validation cohort (Fig 5B). This could be explained by low patient numbers of these rare disease entities and heterogeneous cholestatic liver pathologies in this cohort. To further analyse the origin of observed increase in DPPIV serum activity in vitro experiments on cholangiocytes were performed (data not shown). But no difference on DPPIV mRNA expression was observed 8h after stimulation with TLC, CDCA, INT-747 and TGR5 agonist. Therefore, further in vitro studies with different cell lines (hepatocytes, immune cells) are needed to better understand the observed increase in DPPIV activity.

In summary, there is a strong linkage between increased levels of serum BA, IP-10 and DPPIV activity in HCV patients. In cholestatic HCV patients these changes had impact on peripheral immune cells with higher frequency of CXCR3+ T cells. In cirrhotic patients BA, IP-10 serum levels as well as DPPIV activity was higher and cirrhotic patients showed a trend towards higher frequency of peripheral CD3+CXCR3+ T cells. These observations could be expression of the advance liver pathology and characterize cirrhotic patients as difficult to treat patients with increase susceptibility for infections.

The above described results underline our initial hypothesis that elevated serum BA could contribute to higher DPPIV activity, but the underlying mechanism remains elusive. Since these observations are of clinical importance for the basic understanding of cholestasis with immune modulatory capacity of BA and hepatic inflammation in various liver diseases, further analysis of the underlying mechanism of the described associations is warranted. The question of whether BA *per se* or other mediators retained during cholestasis are causative for increased DPPIV activity remains open and deserves further in vitro investigation.

## Supporting information

S1 Fig **A-B**) In the FU cohort of HCV patients serum IP-10 levels as well as DPPIV serum activity are depicted between patients with and without cholestasis. Mean ± SD. Wilcoxon Mann Whitney test; * = p<0.05. **C-E**) IP-10 serum levels, DPPIV serum activity and BA in cirrhotic patients and patients without cirrhosis. Mean ± SD. Wilcoxon Mann Whitney test; * = p<0.05; ** = p<0.01.
(TIFF)

S2 Fig **A-B**) Taurin-BA and Glyco-BA in the FU cohort in HCV patients and HC. Mean ± SD. Wilcoxon Mann Whitney test. **C-D**) Correlation between serum IP-10 levels and Taurin-BA as well as Glyco-BA in the FU cohort. Spearman Rho as indicated.
(TIFF)

S3 Fig **A)** Three cholestatic patients of the FU cohort showed a trend toward higher CD3+CXCR3+ T cells. Mean ± SD. Wilcoxon Mann Whitney test. **B)** Cirrhotic HCV patients showed a trend towards higher peripheral CD4+CXCR3+ T cells. Mean ± SD. Wilcoxon Mann Whitney test.
(TIFF)

## Abbreviations

BA: Bile acids
BSEP; ABCB 11: Bile salt export pump
CDCA: Chenodeoxycholic acid
DPPIV/CD26: Dipeptidyl peptidase 4
GT: Genotype
HC: Healthy control
HCV: Hepatitis C virus
IP-10/CXCL10: Interferon-gamma-inducible protein-10
MIG/ CXCL9: Monokine induced by interferon-gamma
NASH: Non-alcoholic steatohepatitis
PBC: Primary biliary cirrhosis
Peg-IFN: Pegylated interferon
PSC: Primary sclerosing cholangitis
RBV: Ribavirin
SVR: Sustained virological response
